# Genome-Wide Patterns of Gene Expression during Aging in the African Malaria Vector *Anopheles gambiae*

**DOI:** 10.1371/journal.pone.0013359

**Published:** 2010-10-13

**Authors:** Mei-Hui Wang, Osvaldo Marinotti, Anthony A. James, Edward Walker, John Githure, Guiyun Yan

**Affiliations:** 1 Program in Public Health, University of California Irvine, Irvine, California, United States of America; 2 Department of Molecular Biology and Biochemistry, University of California Irvine, Irvine, California, United States of America; 3 Department of Microbiology and Molecular Genetics, University of California Irvine, Irvine, California, United States of America; 4 Department of Microbiology and Molecular Genetics, Michigan State University, East Lansing, Michigan, United States of America; 5 Division of Human Health, International Centre of Insect Physiology and Ecology (ICIPE), Nairobi, Kenya; Deutsches Krebsforschungszentrum, Germany

## Abstract

The primary means of reducing malaria transmission is through reduction in longevity in days of the adult female stage of the *Anopheles* vector. However, assessing chronological age is limited to crude physiologic methods which categorize the females binomially as either very young (nulliparous) or not very young (parous). Yet the epidemiologically relevant reduction in life span falls within the latter category. Age-grading methods that delineate chronological age, using accurate molecular surrogates based upon gene expression profiles, will allow quantification of the longevity-reducing effects of vector control tools aimed at the adult, female mosquito. In this study, microarray analyses of gene expression profiles in the African malaria vector *Anopheles gambiae* were conducted during natural senescence of females in laboratory conditions. Results showed that detoxification-related and stress-responsive genes were up-regulated as mosquitoes aged. A total of 276 transcripts had age-dependent expression, independently of blood feeding and egg laying events. Expression of 112 (40.6%) of these transcripts increased or decreased monotonically with increasing chronologic age. Seven candidate genes for practical age assessment were tested by quantitative gene amplification in the *An. gambiae* G3 strain in a laboratory experiment and the Mbita strain in field enclosures set up in western Kenya under conditions closely resembling natural ones. Results were similar between experiments, indicating that senescence is marked by changes in gene expression and that chronological age can be gauged accurately and repeatedly with this method. These results indicate that the method may be suitable for accurate gauging of the age in days of field-caught, female *An. gambiae*.

## Introduction

Malaria is transmitted by female mosquitoes of the genus *Anopheles*, kills at least one million people annually, and causes more than 247 million clinical cases each year [1]. Epidemiologically, the most important life history attribute of female *Anopheles* vectors of human malaria is their life span, i.e., their longevity measured in days. The mosquitoes must live long enough to develop infectiousness by bite after having acquired malaria infection from a chronologically earlier blood meal taken from a gametocytemic human, and thereafter incubate that infection to the infective stage [2]. The time interval from acquisition of an infection to the delivery of an infectious bite, called the extrinsic incubation period, is equivalent to the development time of the malaria parasite in the body of the mosquito to the age when salivary glands are infected with sporozoites. The other epidemiologically important life history attributes or population parameters are the range of host choice (human or otherwise), biting rate on humans, and the frequency of biting per unit time (equivalent to the interval in days of the frequency of egg laying, termed the gonotrophic cycle) [3], [4]. The algebraic combination of these variables is called vectorial capacity, operating under the assumption of good vector competence (i.e., physiologic and genetic capacity to support parasite development normally). However, only longevity is exponentially related to the vectorial capacity. Thus, small reductions in longevity cause large reductions in vectorial capacity, independently of the other life history and population parameters, because they limit the mosquito's life span to an age shorter than the extrinsic incubation period (minimally 12 days at field temperatures under typical tropical conditions). None of the other parameters contributing to vectorial capacity have such a sensitive relationship to malaria transmission. Knowledge of the population age structure of mosquito vectors in natural conditions is crucial to the understanding of malaria transmission dynamics. Furthermore, information on mosquito population age structure is critical to the assessment of the impact of environmental changes and vector control measures on malaria transmission. Indeed, the explicit intent and overt effect of the most common means of reducing malaria transmission, by indoor residual spraying or use of insecticide treated bed nets, is in reducing average age in vector populations, thereby delimiting the expectation of infective life of the mosquito vector population. This effect is a result of the cumulative exposure of female mosquitoes to the insecticide treated surfaces or fabrics, resulting in a higher probability of death in older females (who experience more exposures than do younger females).

It has heretofore been impossible to gauge the chronological age of female mosquitoes with accuracy, i.e., to answer the simple question “how old is an adult, female this mosquito in days?” Methods have been restricted to crude categorical means, in particular, examination of changes in conformation and structure of the ovaries that reflect whether a mosquito has not (nulliparous) or has (parous) laid at least one batch of eggs. If she has, it means that she has acquired at least one blood meal and therefore has had at least one opportunity to acquire a malaria infection by biting a gametocytemic, human malaria carrier. But further categorization with this relatively easy method is not possible. The probability of daily survival can be estimated through application of the Davidson formula, which requires knowledge of the duration of the gonotrophic cycle in nature. Ovaries can also be examined for presence of dilatations in the ovariolar ducts but the method is very difficult to accomplish in practice owing to technical limitations. Mosquito age grading tools that enable accurate determination of vectors of various ages are called for helping understand malaria transmission dynamics and for evaluation of the impact of vector control tools. This study utilized microarray technique to determine profiles of gene expression during *A. gambiae* senescence. In sub-Saharan Africa where the malaria burden is the highest, members of the *Anopheles gambiae* complex are the main vector species. Identification of genes associated with aging in *An. gambiae* provide an alternative set of tools for understanding mosquito senescence generally, and measuring chronological age sensitively [5].

Gene expression profiling using microarrays allows for genome-wide examination of changes in the abundance of transcription products during the mosquito aging process. Genome-wide variation in transcription abundance associated with aging has been explored in *Drosophila melanogaster* [6], [7], [8] and *Caenorhabditis elegans* [9], [10]. Aging in the fruit fly is associated with significant expression changes in 2.9–9.7% of all genes [11], [12]. Expression of 22.5% genes are altered significantly by age in *C. elegans* [13]. Age-associated expression changes have been We present here a comprehensive gene expression profiling analysis of the aging process of *An. gambiae*. Because bloodfeeding has a major impact on mosquito gene expression [14], [15], two cohorts of mosquitoes, with and without access to blood meals, were analyzed. This experimental approach is important for the identification of age-grading molecular markers as it allows more robust data analysis and selection of genes that are affected exclusively by age, and not by blood feeding. Seven genes that vary significantly in expression during the aging process and that are not affected by blood feeding were selected and their expression profiles validated by quantitative reverse-transcription real time polymerase chain reaction (qRT-PCR). These genes are candidates for field testing of a transcriptional profiling-based *An. gambiae* age-grading method.

## Results

### Effect of dietary factors on gene transcription

**Figure 1:**
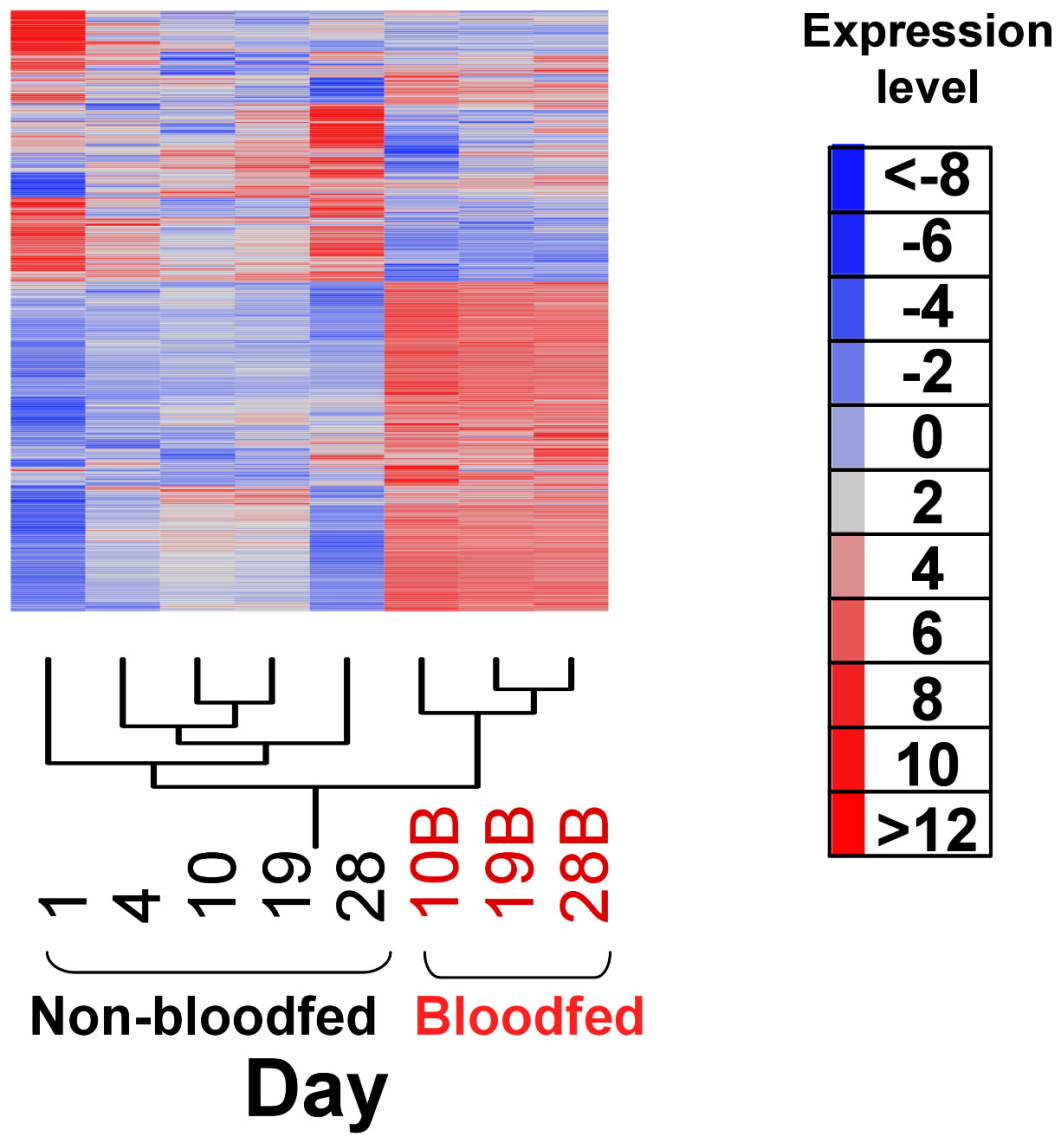
Hierarchical clustering analysis of gene expression in *Anopheles gambiae* adult female mosquitoes of different age groups maintained with access to plant sugar or sugar and rabbit blood. Graphic heat maps represent transcripts expressed differentially among all the age groups (n = 276, p values <0.001). Each row represents data for one probe and each column corresponds to an age group, referred as chronological age (days post adult emergence, pupal-adult ecdysis).

Hierarchical Clustering creates a tree of relationships from gene expression data derived from all age groups and diet status. Hierarchical clustering analysis with all 14,900 probe-sets studied revealed that dietary factors had the largest effect on global gene expression. This is evidenced by separate branching of expression profiles derived from mosquitoes fed with sugar when compared to those fed with blood meals, regardless of their ages. The same pattern of results was found among the different age groups and between blood-fed and sugar-fed mosquitoes when hierarchical clustering analysis was performed with those genes with significant differential expression as identified by the analysis of variance (ANOVA) (Fig. 1). A total of 5,726 (38.4%), 4,687 (31.5%) and 6,624 (44.5%) transcripts were expressed differently in blood-fed mosquitoes at day 10, 19 and 28 post emergence, respectively, when compared to sugar-fed mosquitoes.

### Age-associated changes in gene expression

**Figure 2:**
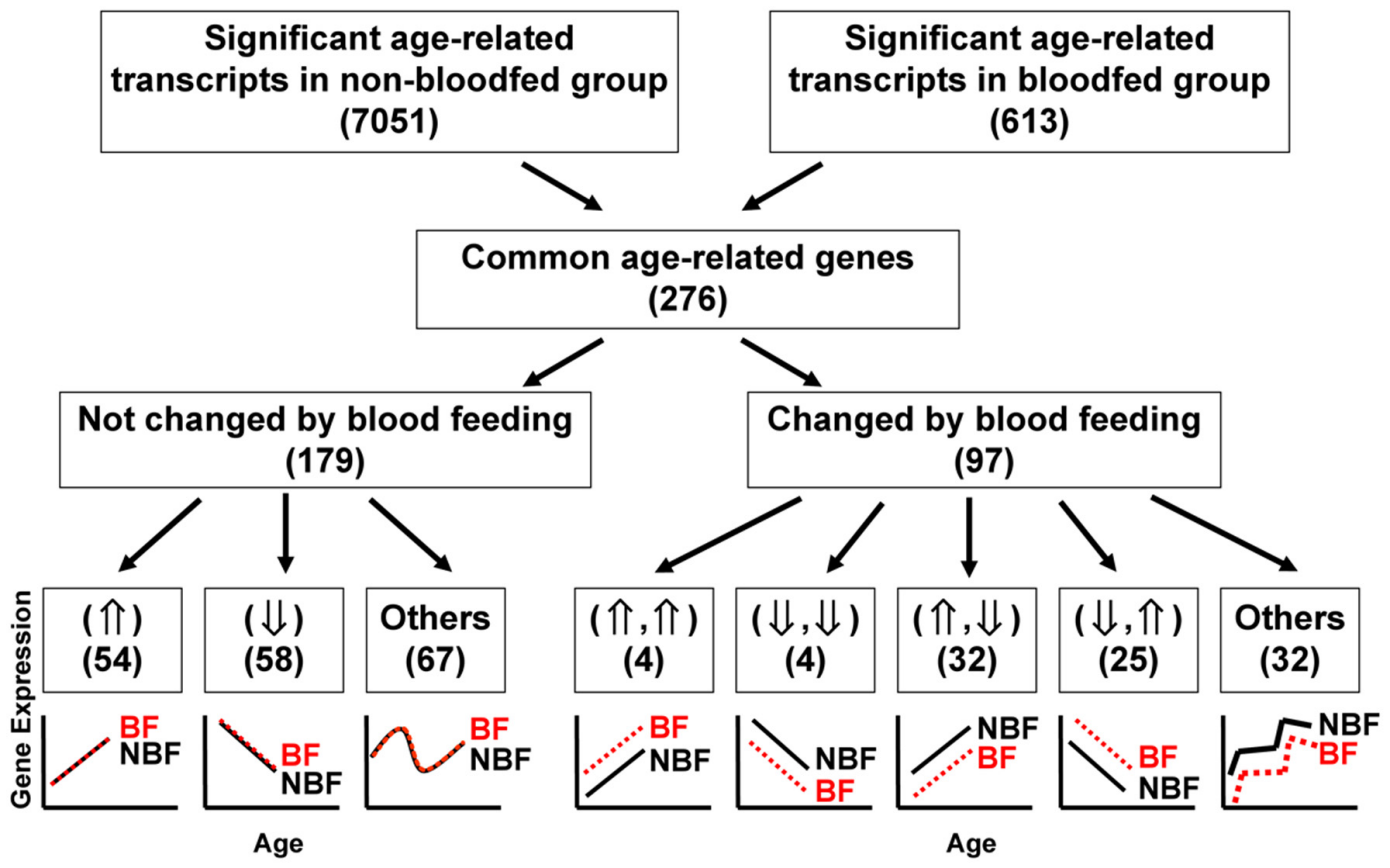
Flowchart of data analysis and identification of age-related transcript accumulation. The numbers in parentheses represent the number of gene-specific transcripts in each category. The ‘⇑’ symbol indicates gene expression increasing with age; ‘⇓’ indicates expression decreasing with age; ‘⇑,⇑’ expression increasing with age and up-regulated by blood feeding; ‘⇓,⇓’ expression decreasing with age and down-regulated by blood feeding; ‘⇑,⇓’ expression increasing with age and down-regulated by blood feeding; and ‘⇓,⇑’ expression decreasing with age and up-regulated by blood feeding. Genes in the ‘⇑’ and ‘⇓’ categories are considered candidates for the development of a gene expression-based mosquito age-grading methodology.

A total of 7,051 transcripts were differentially expressed significantly in at least one pair of age groups from the five age groups that were not bloodfed. Significant differential expression is defined as P≤0.001 as the cut-off criterion after correction for multiple testing using the bloodfed group as mosquitoes at day 1 post emergence showed the largest difference from other age groups in gene expression profiling. A total of 276 transcripts were determined to be expressed differentially in both non-bloodfed and bloodfed mosquitoes (Fig. 2 and Table S1). These common transcripts were divided into two groups based on whether their expression is regulated by blood-feeding (Fig. 2): 179 transcripts were regulated by aging and their accumulation was not influenced by blood feeding (Table S2), and 97 were influenced by both age and blood feeding (Table S3).

Fifty-four of the 179 transcripts regulated exclusively by aging exhibited an increasing monotonic trend of abundance, 58 showed a decreasing monotonic expression, and 67 transcripts had variable patterns of expression with age (Fig. 2). The annotations of these monotonically increasing or decreasing genes are in Table S4.

### Genetic mechanisms of mosquito aging

**Figure 3:**
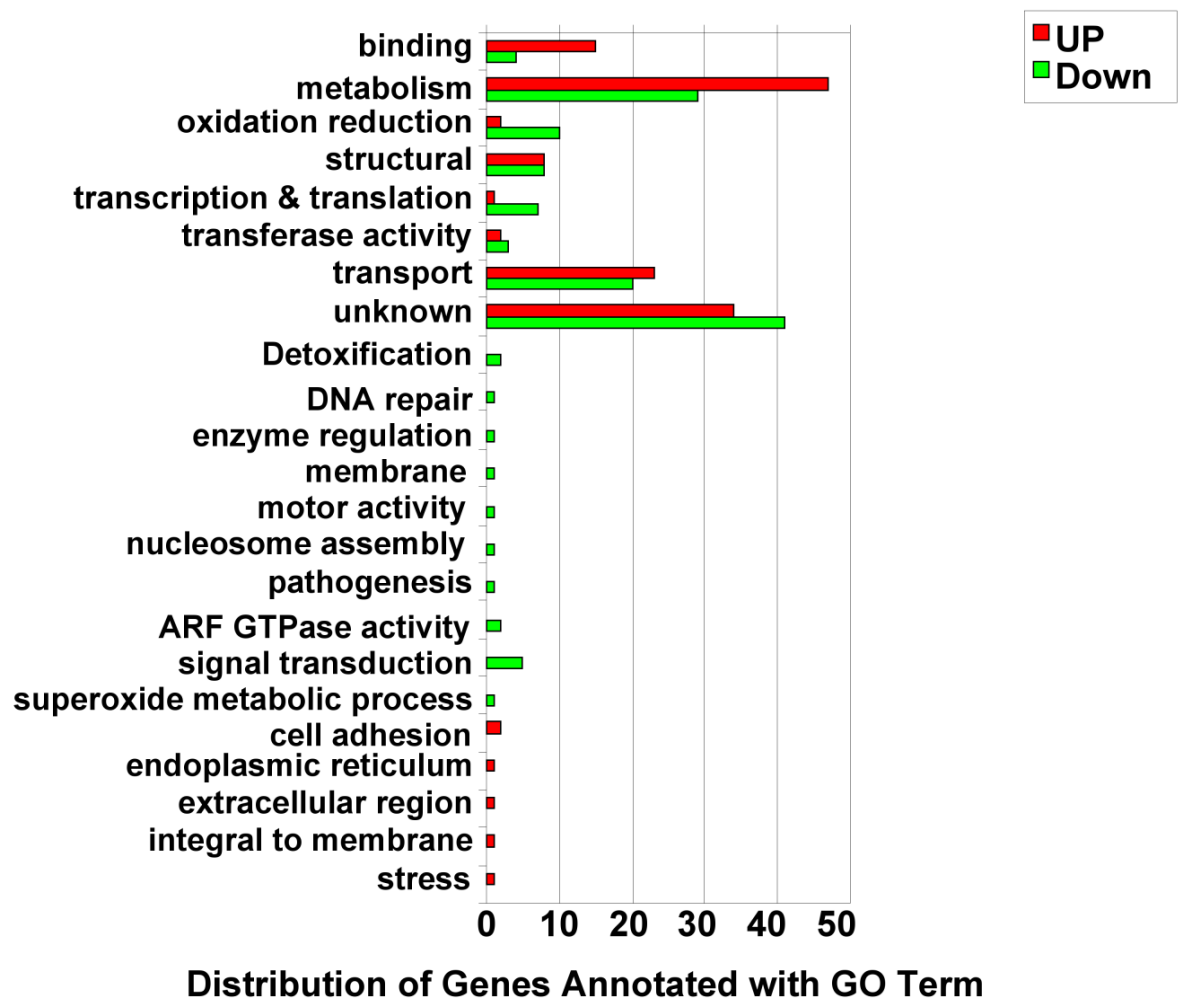
Distribution of the *Anopheles gambiae* genes with expression modulated by aging according to their putative functions. Gene ontology functional class were derived and displayed separately for genes with decreasing (in green) or increasing expression (in red) as the mosquitoes age.

Functional category enrichment based on Gene Ontology (GO) was performed for all significant aging genes (n = 276) in the microarray at up- or down regulated levels when aging. The number of GO terms assigned to genes was compared to all GO-annotated genes in the peptide dataset of *An. gambiae* (Fig. 3). Although many transcripts were encoded unknown function, we still found significant enrichment of GO terms in molecular function including binding, metabolism and transport. Four classes of age-related genes identified in our microarray analysis are particularly noteworthy: 1) stress-response genes, 2) detoxification genes, 3) genes encoding oxidoreductases, and 4) chitin metabolism genes.

#### 1) Stress-response genes

Mosquito aging is accompanied by a gradual decrease in the physiological capacity to adapt to environmental stresses. The fundamental functions of heat shock proteins are molecular chaperoning and cellular repair. It has been proposed that increased protein damage during aging may be exacerbated by a declining heat-shock response, reduced levels of heat-shock proteins, and the resultant loss of protein quality control [16], [17]. The *D. melanogaster hsp70* gene is induced in response to acute heat and oxidative stress and also is up-regulated during normal aging [18]. Expression of *hsp70*-fusion transgenes is partially predictive of drosophila survival under normal and stress conditions. *AGAP004192* encodes a mosquito heat-shock 70 kDa protein (hsp70). Its expression level decreases during the mosquito senescence in animals fed only on sugar, but it is up-regulated by blood meals. This is consistent with the interpretation that the supplements provided by a proteinaceous diet reduce the aging-related stress. *AGAP007347*, exhibits age-dependent expression, increasing in abundance as the mosquitoes age. It encodes a lysozyme (*LysC1*) [19] that hydrolyses carbohydrate molecules in bacterial cell walls. Variations in lysozymes expression have been associated with aging and with immune defense [20], [21].

#### 2) Detoxification genes

Cytochrome P450 genes have a wide range of biological functions, including drug metabolism, detoxification of xenobiotic compounds, electron transport, and synthesis of sterol/fatty acids [20]. The expression of P450 genes varies significantly during the aging process in *Drosophila* [8], [12], [20], rat, human [22] and *C. elegans* [23]. Eight cytochrome P450 genes (AGAP000088, AGAP000818, AGAP008213, AGAP008219, AGAP008358, AGAP009246, AGAP012296, AGAP012296) were up-regulated and had transcription products that increased monotonically with age (Table S4). Five of these (AGAP008219, AGAP000088, AGAP000818, AGAP012296 and AGAP009246) have known *Drosophila* orthologues (Cyp6d4, Cyp4d2, Cyp9f2, Cyp9b2 and Cyp4c3, respectively).

Glutathione S-transferases (GSTs) are detoxification enzymes that insects use to modulate oxidative and chemical stresses, and to metabolize a wide range of hydrophobic toxic compounds such as insecticides and toxic endogenous substrates [24]. Three genes encoding glutathione S-transferases exhibited monotonic changes in expression. AGAP009194 showed decreasing expression with increasing mosquito age, whereas AGAP004163 and AGAP004378 displayed steadily increasing expression. AGAP004378 is orthologous to *D. melanogaster GST D1*, which is known to metabolize insecticides [11], [25]. Older *An. gambiae* exhibit reduced susceptibility to pyrethroid insecticides [26], most likely reflecting the net effects of various detoxification genes during the aging process.

#### 3) Oxidoreductase encoding genes

The aging process involves comprehensive down-regulation of mitochondrial genes, and nearly all components of respiration and organelle function are affected [12], [27]. Ten transcripts (AGAP012394, AGAP012395, AGAP012263, AGAP005499, AGAP006926, AGAP005501, AGAP005323, AGAP007479, AGAP007497 and AGAP004500) were identified that encode oxidoreductases (Table S4), most of which exhibit decreased expression during aging. In particular, AGAP006926 encodes a Rossmann-fold NAD(P)(+)-binding protein, and is orthologous to *D. melanogaster Pdh*, which affects life span in fruit flies [8]. AGAP007479, orthologous to *D. melanogaster* CG7560, encodes a methylenetetrahydrofolate reductase (MTHFR),which was identified to be involved in oxidative stress response in the fruit fly [11]. AGAP012394 encodes a peptide methionine sulfoxide reductase, is orthologous to *D. melanogaster Eip71CD*, and is a conserved protein associated with aging processes in human and invertebrates [28], [29]. AGAP007497 encodes a copper/zinc superoxide dismutase (SOD) and has increasing abundance as the mosquito ages. SOD is an important antioxidant defense in nearly all cells exposed to oxygen. Although the precise role of antioxidant defense in aging remains to be understood, there is a large amount of evidence correlating increased oxidative damage with aging [20], [30], [31].

#### 4) Chitin metabolism genes

Changes in chitinase enzymatic activity has been associated with *D. melanogaster* aging [8], [20]. Eleven genes encoding cuticle proteins or protein involved with chitin metabolism or containing chitin binding domains exhibit either increasing (AGAP011615, AGAP011616, AGAP011322 and AGAP008244) or decreasing (AGAP006001, AGAP006370, AGAP006829, AGAP003261, AGAP009869 and AGAP009790) transcript accumulation during mosquito aging (Table S4). The impact of these variations in expression on cuticle integrity has not been investigated, however the expression of these genes is not influenced by blood feeding, and therefore they are candidate markers for age-grading.

#### 5) Signal transduction genes

Signal transduction converts a stimulus to a receptor, and ends with a change in cell function. For example, the insulin signaling cascade plays a central role in regulating immune and oxidative stress responses that affect the life spans in nematode and fruit fly. In mosquitoes, it has demonstrated that molecules from the invading parasite and the blood meal elicit functional responses in female mosquitoes that are regulated through the insulin signaling pathway or by cross-talk with interacting pathways [32]. In this study, we identified 5 down regulated genes (AGAP006088, AGAP008593, AGAP008302, AGAP002156 and AGAP008301) related to signal transduction when aging. Through GO molecular function, AGAP008593 is identified as insulin-like growth factor binding protein and process signal transduction. The details of signal transduction and its relevant pathways present significant challenges for future research, but will increase our understanding of mosquito of the conservation of insulin signaling and aging regulatory.

### Semi-quantitative and quantitative gene amplification analyses of expression profiles

**Figure 4:**
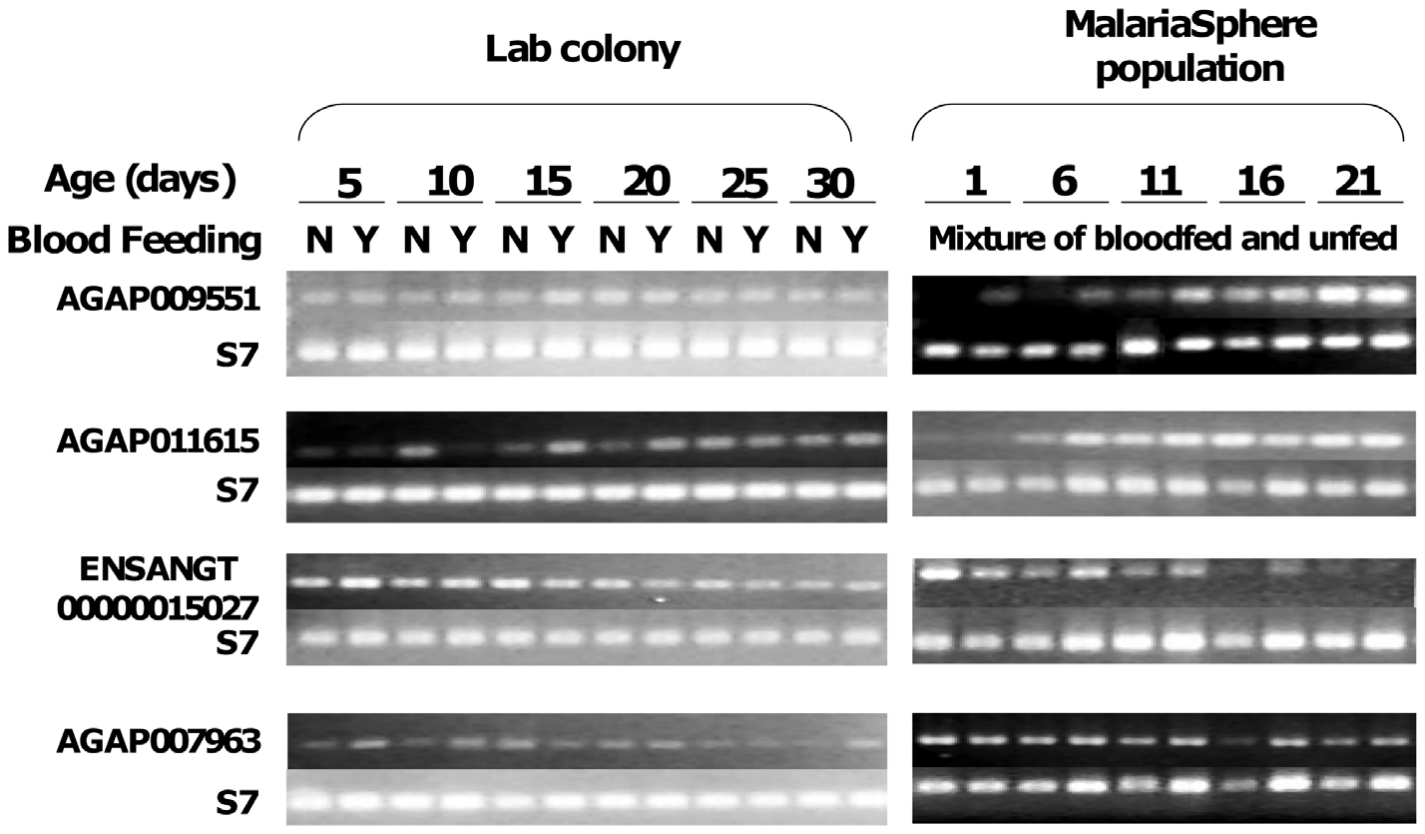
Semi qRT-PCR validation of gene expression patterns of four selected genes reveal consistent gene expression variations between two strains maintained in distinct environments. *Anopheles gambiae* G3 strain mosquitoes raised in a local insectary, at UCI, CA, USA (Lab colony) and Mbita strain mosquitoes maintained in the MalariaSphere in western Kenya (MalariaSphere populations) were used as the source of RNA. RNA extraction and qRT-PCR conditions were identical to all samples. Age refers to chronological days post pupal emergence in adult females.

**Figure 5:**
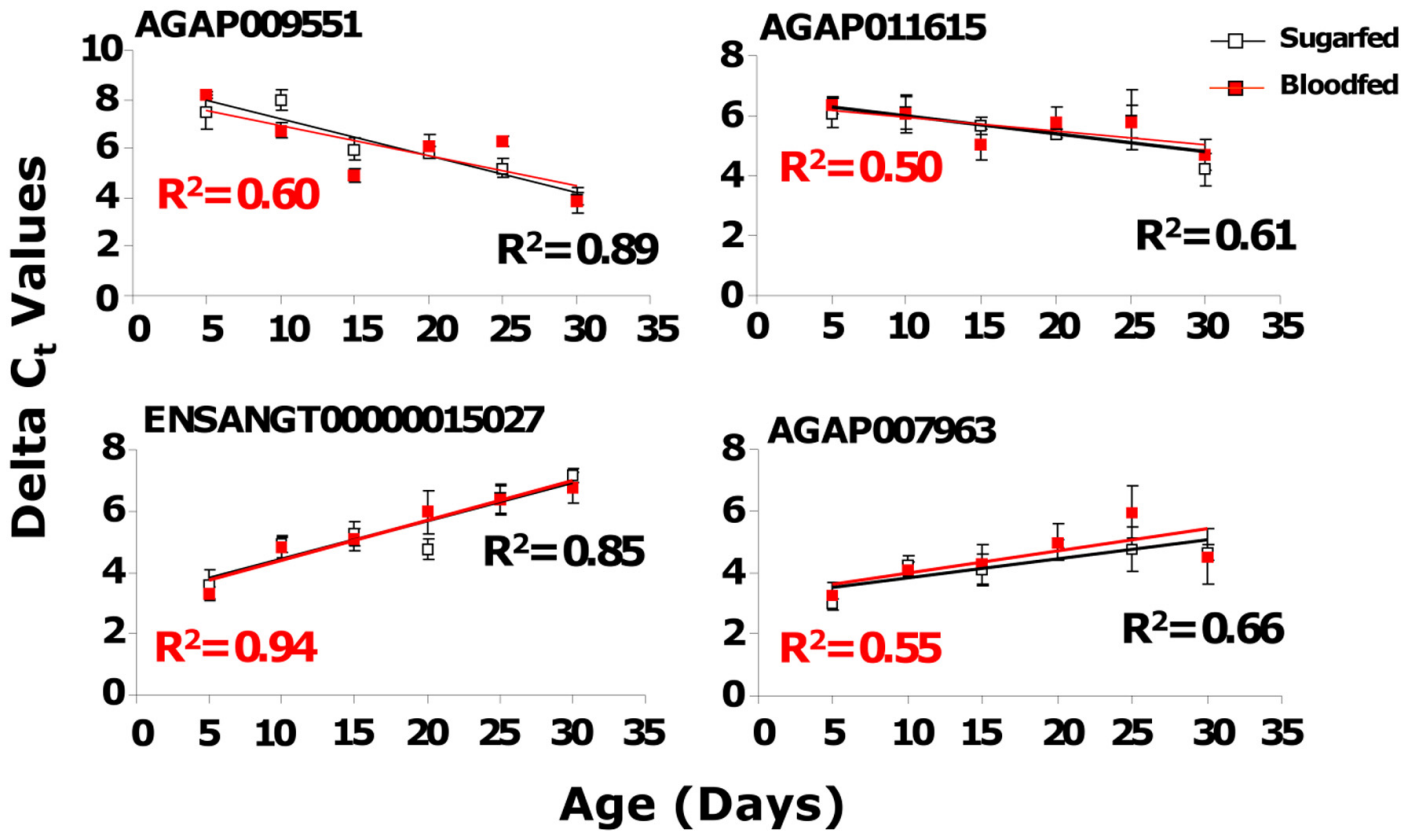
Validation of variations in gene expression by qRT-PCR. Regression analyses were performed correlating chronological ages and transcript abundance for 4 of the age-grading candidate genes. Mosquito chronological age is the days post pupal emergence.

Semi-quantitative gene amplification of the products of four out of seven selected genes (AGAP009551, AGAP011615, ENSANGT00000015027 and AGAP007963) was performed with mosquitoes derived from our lab and the semi-field MalariaSphere in western Kenya (Fig. 4). These analyses confirmed the monotonic expression changes with mosquito age as predicted by the microarray results. Similarly, quantitative gene amplification established monotonically decreasing or increasing expression in the four out of seven selected genes (Fig. 5). For each age group (for both NBF and NBF), 3 individual female mosquitoes were tested and repeated twice (all p<0.05). Therefore, the microarray results were verified independently by semi-quantitative and quantitative RT-PCR methods.

### Comparison of age-regulated genes in *An. gambiae* and *D. melanogaster*

The *An. gambiae* age-related gene expression data were compared to similar results from *D. melanogaster* [6], [8], [20], [33]. Table 1 lists those mosquito genes that are orthologous to those of the fruit fly. The NCBI HomoloGene database was utilized to identify orthologous genes regulated during aging in *D. melanogaster*. Ten pairs of *An. gambiae* and *D. melanogaster* homolog genes (*CG11159* [34], *Cyp6d4* [8], [35], *Cyp4d2* [8], *Cyp9f2* [36], *Eip71CD* [28], [29], CG7798 [20], *Cyp9b2*, *Cyp4c3*, *GstD1* [8], [25] and *Pbprp2* [8], [37]) show increasing abundance with age in both the mosquito and fruit fly (Table 1). Alternately, seven genes (*Ect3* [38], *Pdh* [8], *SelD* [39], *Taz* [40], *Scp1* [11], [41], *pgant6* [8], [42] and *Cpr49Ae* [8], [11]), show decreasing abundances in both species. Four genes, *Git* [8], *pug* [43], *crq* [8] and *Hsc70-3* [43], exhibit opposite trends in the aging-expression relationship between *An. gambiae* and *D. melanogaster*.

**Table 1:** *Anopheles gambiae* genes related to *Drosophila* aging.

Probe Set ID	Age effect*	Bloodfeeding effect†	Functional group	Functional assignment	Best match to DMPROT database
Ag.2L.458.0_CDS_a_at	↑	no	Cellular Process	C-type lysozyme	*CG11159* ‡
Ag.3R.713.0_CDS_s_at	↑	no	Detoxification	Cytochrome P450	*Cyp6d4*
Ag.X.268.0_CDS_a_at	↑	no	Detoxification	Cytochrome P450	*Cyp4d2*
Ag.X.7.0_CDS_at	↑	no	Detoxification	Cytochrome P450	*Cyp9f2*
Ag.3L.178.0_CDS_s_at	↑	no	Metabolism	Peptide methionine sulfoxide reductase	*Eip71CD* ‡
Ag.2L.1715.0_CDS_at	↑	no	Regulation	Putative GTPase activating protein for Arf	*Git* ‡
Ag.2L.29.0_CDS_a_at	↑	no	Response to Stress	Lysozyme precursor	*CG7798*
Ag.2R.1515.1_CDS_a_at	↓	no	Cellular Processes	Autophagic cell death	*Ect3*
Ag.2R.365.0_CDS_a_at	↓	no	Metabolism	Formate-tetrahydrofolate ligase	*pug* ‡
Ag.2L.1721.0_CDS_at	↓	no	Metabolism	Rossmann-fold NAD(P)(+)-binding proteins	*Pdh*
Ag.3L.433.0_CDS_at	↓	no	Metabolism	PurM-like; AIR (aminoimidazole ribonucleotide) synthase related protein.	*SelD*
Ag.2L.284.0_CDS_at	↓	no	Metabolism	Acyltransferase	*Taz* ‡
Ag.3R.169.0_UTR_s_at	↓	no	Transport	EF-hand, calcium binding motif	*Scp1* *
Ag.3L.353.0_CDS_s_at	↑	↓	Detoxification	Cytochrome P450 (Fragment)	*Cyp9b2*
Ag.3R.739.0_CDS_at	↑	↓	Detoxification	Cytochrome P450	*Cyp4c3*
Ag.2R.46.0_CDS_at	↑	↓	Metabolism	GST_C family, Class Delta and Epsilon subfamily, cellular detoxification	*GstD1*
Ag.2R.70.0_CDS_at	↑	↓	Transport	Pheromone-binding protein	*Pbprp2*
Ag.UNKN.1744.0_CDS_s_at	↓	↑	Cellular Processes	Autophagic cell death	*crq* ‡
Ag.3L.1690.0_CDS_at	↓	↑	Metabolism	Oligosaccharide biosynthesis	*pgant6*
Ag.2R.399.0_CDS_a_at	↓	↑	Response to Stress	Heat shock 70 kDa protein	*Hsc70-3* ‡
Ag.3R.644.0_CDS_a_at	↓	↓	Structural	Chitin_bind_4; Insect cuticle protein	*Cpr49Ae* ‡

Genes with expression variation associated with aging are conserved among *Anopheles gambiae* and *Drosophila melanogaster.*

*Age effect:” **↑** ” indicates monotonically increasing expression with mosquito age, and “**↓**“ monotonically decreasing expression.

† Bloodfeeding effect: “no” indicates no effects by bloodfeeding on gene expression, “ **↑** ” pp-regulation by blodfeeding, and “ **↓** “ down-regulation by bloodfeeding.

‡ *Drosophila melanogaster* homologous gene name.

### Identification of candidate genes for *An. gambiae* age grading

Five criteria were used to select candidate genes for mosquito age grading: 1) expression is not affected by blood feeding, 2) the genes were not documented as being associated with insecticide resistance, 3) the expression of the candidate genes changes monotonically with age, 4) known age-dependent genes are present in other insects (e.g., orthologous to *D. melanogaster* age-related genes), if such information is available, and 5) the expression of the candidate genes exhibits the highest-fold changes between young and old mosquitoes. Seven candidate genes (AGAP009551, AGAP011615, AGAP002827, AGAP005501, AGAP009790, AGAP007963 and ENSANGT00000015027), were identified based on these criteria, three with monotonically increasing and four with monotonically decreasing expression during aging (**Table 2**). In particular, AGAP007963 encodes a calcium-binding domain and is homologous to *D. melanogaster Scp1* and *Aedes aegypti* Ae-15848, both of which show age-dependent expression [5], [11]. In yeast, the deletion of a single gene, encoding the actin bundling protein Scp1, leads to a reduced production of ROS and a highly significant increase in longevity [44]. It suggests that Scp1p plays a role in regulating the dynamics of the actin cytoskeleton in yeast to trigger cell death at an appropriate time, and that loss of Scp1p results in increased actin dynamics and an increase in longevity. However, there are no significant experiments done or showed the similar phenomena in fruit flies or mosquitoes. A Spearman correlation analysis found highly significant correlations between expression value and mosquito chronological age (measured in days post pupal emergence) for all seven genes (Table 2), with R^2^ value ranging from 0.5 (P<0.01) to 0.93 (P<0.01). This demonstrates the potential of using the candidate genes as biomarkers for *An. gambiae* adult mosquito age grading.

**Table 2:** List of genes selected for *Anopheles gambiae* mosquito age grading.

Gene/Probe ID	qRT-PCR primerForward primer (5′-3′)Reverse primer (5′-3′)	Size	Age effect*	Blood feeding effect†	Functional assignment	Fold changes‡	Correlation coefficient¶
AGAP009551	TGCCGATTAAGATTCCCAAC TTCAAGGAGTTCCTGCTCGT	83bp	↑	no	Sulfotransferase domain	4.2	0.89
AGAP011615	GTCTTCCAACCGGAGGTACA CTCCTCTGGACCGGATGTTA	81bp	↑	no	Chitin binding Peritrophin-A domain	3.28	0.61
AGAP002827	ATTGGAGCGTACGCGGTTA CCAGCTAGCAGCAATCCGTA	83bp	↑	no	Synaptic vesicle membrane	3.49	0.50
AGAP005501	ACGTGATCGACACGAATCTG GCACACCACTGTTACCGATG	285bp	↓	no	Rossmann-fold NAD(P)(+)-binding proteins	−3.95	0.65
ENSANGT00000015027	GTCATCTTCTCGCGGTTAGC CAGTCCAAGCGTGGTATCCT	185bp	↓	no	Actin activities	−5.37	0.93
AGAP009790	TCCTAGTGCTCGTCAGTGTGA GTTCGCCGGTTTAGCTCATA	82bp	↓	no	Chitin binding Peritrophin-A domain	−4.66	0.52
AGAP007963	GATGCCACCCTTCTTGTTGT AATTCAAACAGGCCGTCAAG	239bp	↓	no	EF-hand, calcium binding motif	−4.33	0.66

*Age effect: “↑” and “↓” indicate gene expression monotonically increases decreases with age.

† Blood feeding effect: “no” stands for no significant effect of blood feeding on gene expression.

‡ Fold changes: variation in transcript accumulation between 1-day and 28-day old female mosquitoes, regardless of blood their feeding diet calculated from microarray data.

¶Correlation coefficient: Spearman correlation between gene expression values and mosquito ages in laboratory colonized mosquitoes.

## Discussion

Precise determination of mosquito population age structure under natural conditions is fundamental to the understanding of malaria transmission potential. One of the most important variables in vectorial capacity is mosquito life span, because vectorial capacity is strongly influenced by vector longevity through an exponential, power and logarithmic relationship when considered epidemiologically. Therefore, a mosquito population with a relatively long average life expectancy has a much greater capacity to transmit malaria than does a population of identical attributes but with a shorter average life expectancy. Population age structure of mosquito vectors in natural conditions also is crucial to the assessment of the impact of environmental changes and vector control measures on malaria transmission. The current methods for mosquito age grading, i.e., ovary tracheation and dilatation [45], [46], [47], [48], fluorescent pigment quantity [49], cuticular hydrocarbon abundance [50], [51], and near-infrared spectroscopy [52], are problematic in determining the age of old mosquitoes by having substantial variability or limited application to multiple species. Cook et al. [5] tested the idea of using gene transcriptional profiles for age grading in *Ae. aegypti* with genes orthologous to those that show significant changes in expression with age in *D. melanogaster*. They demonstrated that the technique based on gene expression changes can reliably estimate the age of *Ae. aegypti* mosquitoes. However, the precision of the determinations may not be good enough to measure impact on virus transmission mediated by this species. The present genome-wide scan of gene expression in *An. gambiae* provides information on the candidate biomarkers for developing transcription-based age grading method.

We used stringent criteria in our selection of candidate genes as biomarkers for *An. gambiae* age grading that eliminate the impact of bloodfeeding and exclude most P450 gene (detoxification) which are related to insecticide resistance. Those genes whose transcripts are related to bloodfeeding and insecticide resistance are not suitable for age grading purposes, because female mosquitoes pass through several gonotrophic cycles in their lifespan, and are subjected to repeated exposure to insecticides as insecticide-impregnated bed nets or indoor residual spray currently constitute the most important malaria control methods. Other criteria for our age-grading candidate genes selection include known functions in aging in other insects, and monotonically increasing or decreasing expression with age. Based on these criteria, we identified seven candidate genes that were highly significantly correlated with mosquito age in colonized mosquitoes in laboratory conditions, and we were able to rule out functional genes that might mask senescence processes due to cyclic physiological events. We are currently evaluating the suitability and sensitivity of these candidate genes for age-grading in field-collected *An. gambiae* mosquitoes in Africa. Further, we are determining the minimum number of markers required for targeted accuracy and sensitivity in *An. gambiae* age-grading.

This study has shown that biomarkers for aging can be identified by microarray studies and can be applied to adult female *An. gambiae* laboratory and semi-field population. The results show that mosquitoes do indeed senesce, and that knowledge of the process can be used to estimate chronological age. The results provide a means for evaluating age structure of field populations. Gene transcription of mosquito age grading can be made more efficient if environmental factors are considered as well, in particular, temperature, because temperature is an important exogenous factor influencing metabolite rate [53], [54]. Therefore we suggest that the temperature adjustment for transcriptional aging grading should be measured to determine wild caught mosquitoes accurately.

## Materials and Methods

### Ethics Statement

The animal usage was approved by the Committee of Institutional Animal Care and Use Committee of the University of California, Irvine. The regulation for the review committee of laboratory animal welfare and ethics and protocol for the review on laboratory animal welfare and ethics, Institutional Animal Care and Use Committee of the University of California, Irvine, were followed.

### Laboratory mosquito rearing

*Anopheles gambiae* G3 strain, obtained from Malaria Research and Reference Reagent Resource Center (http://www.malaria.mr4.org), was used in the microarray analysis. Mosquitoes were reared in a walk-in insectary regulated at a relative humidity of 75%, temperature of 26°C, and 12∶12 hour light-dark cycle. Mosquito larvae were reared in trays of 100 first-instar larvae per liter of water, and fed with a mixture of Tetramin® (Tetra Werke, Melle, Germany) fish food and yeast. Adult mosquitoes were maintained in cages with 250 mosquitoes per cage of size 128 oz, with an equal sex ratio. Adults had access to raisins and cotton balls saturated with deionized water. Mosquito colonies were exposed to anesthetized rabbits for blood feeding. A total of eight 128-oz cages of mosquitoes (n = 2000) were set up for mosquito specimen preparation for the microarray experiment. The microarray analysis used female adult mosquitoes collected at days 1, 4, 10, 19 and 28 post pupal emergence. Day 1 and day 4 age groups were not bloodfed. From day 4, the mosquitoes were split into two groups, one group mosquitoes were maintained on plant sugar (raisins), but not with any blood source throughout the study, and the other were blood fed every five days. For each cage at each age group, 3 female mosquitoes were collected for the microarray study. Therefore, this design allows comparison of gene expression between different age groups and between mosquitoes maintained with blood and plant sugar.

### Malariasphere mosquito sampling in western Kenya

*Anopheles gambiae* reared in semi-natural MalariaSphere conditions in Mbita Point Field Station of International Centre of Insect Physiology and Ecology were used to determine the correlation between mosquito age and expression of candidate genes. MalariaSphere is a simulated ecosystem for semi-field studies of anopheline mosquitoes [55]. It contains near-natural breeding sites, a local traditional-style house, and different types of food crops and indigenous wild plants to mimic the natural environment of adult *An. gambiae*. *Anopheles gambiae* held in MalariaSphere can successfully complete their entire life cycle and all the major life-history behaviors (mating, sugar feeding, oviposition and bloodfeeding) within the enclosure [55], [56]. The temperature and relative humidity inside MalariaSphere is very similar to the natural ambient conditions [57]. Freshly emerged *An. gambiae* (500 females and 500 males) were released into MalariaSphere with access to human volunteers sleeping in the MalariaSphere twice a week. The human subject protocol was approved by the Institutional Review Board of UC-Irvine. Ten to fifteen mosquitoes were collected every 5 days from day 1 until no female adults were available for collection. All mosquitoes were preserved in RNAlater® (Sigma) and maintained in −20°C freezer before transporting to UCI and then at −80°C before RNA extraction.

### RNA sample preparation and hybridization to Affymetrix GeneChip® arrays

Isolation of total RNA for mosquitoes used in the microarray study followed the protocol recommended by Affymetrix Inc. (Santa Clara, CA). Briefly, three female mosquitoes per age group per replicate were homogenized in TRIzol Reagent (Gibco BRL Life Technologies, Rockville, MD, USA) and purified further by passing through a spin column (Invitrogen, Carlsbad, CA). Total RNA was quantified and adjusted to a final concentration of 10 ng/µL. All starting total RNA samples were quality-assessed prior to beginning target preparation and processing steps by running a portion of each sample (typically 25–250 ng/well) onto a RNA Lab-On-A-Chip (Caliper Technologies Corp., Mountain View, CA) that was evaluated on an Agilent Bioanalyzer 2100 (Agilent Technologies, Palo Alto, CA). Using the NuGEN Ovation RNA Amplification System V2 (NuGEN Technologies, San Carlos, CA), first-strand cDNA was synthesized from the poly(A)+ mRNA present within the isolated total RNA (20 ng total RNA starting material used in each sample reaction). Fragmentation of the mRNA within the resulting cDNA/mRNA complexes created priming sites for DNA polymerase to synthesize DNA complementary to the first-strand cDNAs. The resulting double-stranded cDNAs have a unique DNA/RNA heteroduplex at one end. These double-stranded cDNAs templates were used to perform linear isothermal DNA amplification (SPIA, NuGEN Technologies, San Carlos, CA). 3.75 µg of the resulting amplified cDNA material for each sample first was fragmented and subsequently end-labeled using the NuGEN FL-Ovation cDNA Biotin Module V2 (NuGEN Technologies, San Carlos CA). 2.2 µg of the resulting fragmented, biotin-tagged cDNA was placed into a hybridization cocktail (220 µL), with 200 µL actually hybridized at 45°C with rotation for 18 hours (Affymetrix GeneChip Hybridization Oven 640) to probe sets present on an Plasmodium/Anopheles Genome Array (Affymetrix, Santa Clara, CA). The Plasmodium/Anopheles Genome arrays were washed and then stained (SAPE, streptavidin-phycoerythrin) on an Affymetrix Fluidics Station 450 using the FS450_0004 fluidics protocol, followed by scanning on an Affymetrix GeneChip 3000 Scanner 7G. The results were quantified and analyzed using Expression Console ver.1.1 software (Affymetrix) and the PLIER Algorithm default values (Quantification Scale: Linear; Quantification Type: Signal and Detection P-Value; Background: PM-GCBG; Normalization Method: Sketch-Quantile). RNA processing and hybridization were performed at the DNA and Protein MicroArray Facility of UC-Irvine. Four independent replicates were conducted at each age group except Day 28 non-bloodfed group in which 3 independent replicates were used. Therefore, a total of 31 arrays were used in this study.

### Quantitative RT-PCR (qRT-PCR) and Semi qRT-PCR

Total RNA was extracted from individual mosquitoes using the PureLink™ RNA Mini Kit (Invitrogen), and treated with DNase I. RNA samples free of genomic DNA were used for reverse transcription, using iScriptTM cDNA synthesis Kit (Bio-RAD, Hercules, CA). qRT-PCR assay were developed for each *An. gambiae* candidate gene to quantify their expression. PCR primers for each candidate gene were designed using Primer 3.0 (http://www.broad.mit.edu/cgi-bin/primer/primer3_www.cgi) (Table 2), based on the corresponding cDNA sequences obtained from Anobase (http://www.anobase.org/) and the AnoXcel database (http://www.anobase.org/) [58].

The qRT-PCR was performed using the SYBR Green Master Mix (Fermentas Inc., Glen Burnie, MD) on the MJ Research DNA Engine Opticon RT-PCR System (Bio-RAD). qRT-PCR reaction used the following program: one cycle of 95°C for 5 min, followed by 44 cycles of 94°C for 30 s, 55°C for 30 s and 72°C for 45 s and extension at 72°C for 5 min. Ribosomal protein S7 was used as the standard for expression normalization for each gene and each age group. The qRT-PCR assays were conducted in triplicate, and the average value of the triplicate was used for each gene at each age group. Semi qRT-PCR also used S7 gene primers and amplification for normalization. All reactions were carried out in the linear range of PCR amplification cycles, as determined for each gene, to prevent saturation bias. RT-PCR products (6 µl) were loaded onto ethidium-bromide stained agarose gels (1.0% w/v) and visualized using gel documentation system.

### Data analysis

The signal values of microarray hybridization were normalized by the probe logarithmic intensity error (PLIER) algorithm. To identify the genes differentially expressed among the five age groups examined, one-way ANOVA was conducted within non-bloodfed group and bloodfed group with p-values ≤0.001 as the cut-off criterion as differentia expression, after correction for multiple testing using a Benjamini and Hochberg false discovery rate of 0.01. After identifying the common aging genes among bloodfed and non-bloodfed age groups, these genes were further tested to determinate the effect of bloodfeeding through pair-wise comparison. A hierarchical clustering was employed to examine the degree of similarity of gene expression among all age groups. The clustering method is based on an agglomerative hierarchical clustering procedure, each observation begins in a cluster by itself and pairs of clusters are merged as one moves up the hierarchy [59]. The JMP® Genomics 4.0 software package was used to perform these statistical analyses (SAS Institute Inc., Cary, NC). Spearman's regression analysis was conducted to determine the correlation coefficient between gene expression value obtained from qRT-PCR and mosquito's chronological age.

### Data deposition

All data sets have been deposited in the Gene Expression Omnibus, http://www.ncbi.nlm.nih.gov/geo/query/acc.cgi?token=vfebzaukiucgotq&acc=GSE19756. (accession nos. GSE19765 and GSM493444-493474).

## Supporting Information

Table S1 Microarray-derived expression level of 276 transcripts of the proliferation aging in both non-bloodfed and bloodfed female An. gambiae.
(0.34 MB XLS)

Table S2 Among 276 transcripts, 179 transcripts were regulated by aging and their accumulation was not influenced by blood feeding.
(0.16 MB XLS)

Table S3 Among 276 transcripts, 97 transcripts were influenced by both age and blood feeding.
(0.09 MB XLS)

Table S4 The annotations of monotonically increasing or decreasing transcripts when aging.
(0.08 MB XLS)
